# Les fractures distales de la clavicule type II de Neer: plaque à crochet versus brochage transacromiale

**DOI:** 10.11604/pamj.2015.20.105.4532

**Published:** 2015-02-04

**Authors:** Atif Mechchat, Mohammed Elidrissi, Mohammed Shimi, Abdelhalim Elibrahimi, Abdelmajid Elmrini

**Affiliations:** 1 Department of Orthopaedics and Trauma Surgery B4, UH Hassan II, Fez, Morocco

**Keywords:** Clavicule distale, fracture instable, plaque à crochet, brochage, distale clavicule, unstable fracture, hook plate, pin

## Abstract

Cette étude a été menée afin de faire une comparaison entre deux techniques chirurgicales différentes: la plaque à crochet et l'embrochage dans les fractures instables du quart externe de la clavicule. Nous avons mené une étude prospective entre 2009 et 2013, incluant deux groupes de patients: un premier groupe de 14 patients traités par plaque à crochet par voie d'abord antéro-inférieure, un second de 12 patients traités par brochage. Tous les patients ont été hospitalisés 24 h après la chirurgie et ont été suivi pendant 1 an. Nous avons comparé les résultats des deux techniques en étudiant: le temps opératoire, le saignement, délai de consolidation, la douleur et la fonction selon le score de constant. L'analyse statistique des résultats fonctionnels et radiologiques a montré la supériorité d'une technique par rapport à l'autre; ainsi l’âge moyen global était de 32,6 ans (+/- 13,7), le sex-ratio (H/F) était de 1. Le temps opératoire moyen est de 35 min pour la plaque à crochet contre 45 minutes pour le brochage, le délai moyen de consolidation était de 6,1 (+/-0,7) semaines dans le groupe traité par plaque vissée, et de 6 (+/-0,7) semaines dans le groupe traité par embrochage (p = 0,5), le score de Constant absolu moyen était respectivement de 86 (+/-10,4) et de 90,92 (+/-2,5) (p = 0,04). L'analyse uni variée a montré une association statistiquement significative entre les paramètres d’évaluation et les deux techniques chirurgicales étudiées. Par conséquent, l’étude a noté la supériorité de la plaque à crochet contre l'embroche dans les fractures instables du quart externe de la clavicule.

## Introduction

Les fractures instables du quart externe de la clavicule instable ou type II de Neer ne représente que 12% à 15% des fractures de la clavicule. La séparation de la clavicule du complexe ligamentaire coracoclaviculaire ainsi que le jeu musculaire du membre supérieur sont d'autant des facteurs d'instabilité de la fracture ce qui impose la nécessité d'un traitement chirurgical, autrement le taux de pseudarthrose avoisine les 20% en cas de traitement orthopédique [1, 2]. Plusieurs techniques chirurgicales ont été rapportées dans la littérature [3–6] et aucune n'est considérée comme un gold standard. Plusieurs études ont rapporté chacun l'intérêt d'une technique chirurgicale en particulier mais peu d’écrits rapportés la supériorité d'une technique par rapport à une autre [7, 8]. L'objectif de cette étude est établir une comparaison entre la plaque à crochet et le brochage transacromiale dans les fractures distales de la clavicule type II de Neer.

## Méthodes

Il s'agit d'une étude prospective réalisée au service de chirurgie ostéoarticulaire, entre 2009 et 2013, incluant tous les patients avec une fracture type II de Neer. Tous les patients de plus de 45 ans ou moins de 20 ans, ou avec un antécédent de traumatisme ou de fracture simultanée du membre supérieur ou de lésions vasculaire ou nerveuse ou des patients non coopérants ont été exclus de notre étude. 14 patients sont traités par une plaque à crochet et 12 par un brochage transacromiale.

**Figure 1:**
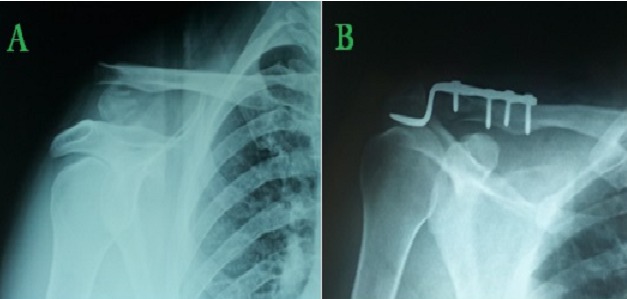
Homme de 34 ans avec une fracture distal de la clavicule type II Neer bénéficiant d'une ostéosynthèse par plaque à crochet. (A) radiographie préopératoire. (B) contrôle radiographique à 6 mois

Tous les patients sont hospitalisés 24 h en postopératoire pour l'antibioprophylaxie et l'analgésie. Dans le groupe plaque à crochet nous avons eu recours à l'anesthésie générale en position demi assise en pratiquant une incision antéro-inférieur suivant le relief de la clavicule, avec réduction transitoire de la fracture par une broche après repérage de l'articulation acromioclaviculaire et stabilisation par une plaque à crochet 6 ou 8 trous 3,5 mm à compression dynamique (DCP) dont le crochet est placé sous l'acromion (Figure 1). Une écharpe d'immobilisation du membre supérieur pendant 1 mois avec une rééducation passive et active dès la sédation des phénomènes inflammatoires. L'ablation de la plaque est réalisée dans une moyenne de 6 mois sous anesthésie générale de courte durée.

**Figure 2:**
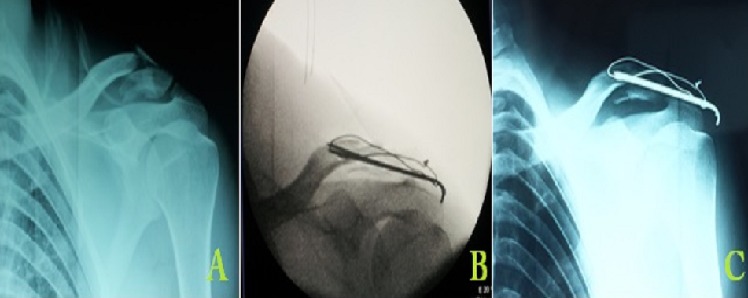
Homme de 28 ans avec une fracture type II de Neer ayant bénéficié d'un brochage tranacromiale renforcé par un hauban au fils d'acier. (A) radiographie préopératoire montrant une fracture type II de Neer. (B) contrôle per opératoire montrant la réduction de la fracture. (C) contrôle radiographique à 4 mois au moment de l'ablation de matériel

Dans le groupe brochage nous avons utilisé un brochage intra-articulaire renforcé par un hauban par un fils d'acier 1,4 (Figure 2). Une immobilisation par une écharpe du membre supérieur est préconisée pendant 4 semaines. Une mobilisation douce et progressive est débutée après sédation de la douleur. L'ablation de matériels est réalisée à 5 mois en moyenne sous anesthésie locale. Tous nos patients sont suivie sur une durée moyenne de 1 an au rythme de 3, 6,12 semaines puis 6mois et 1 an.

### Variables

Le recueil des données a été réalisé à partir d'une fiche d'exploitation contenant l’âge, le sexe, le côté atteint, le mécanisme du traumatisme, le stade radiologique de la fracture, le temps opératoire, le saignement, le délai de consolidation, l’évaluation fonctionnelle, la réduction anatomique et les complications postopératoires. L’évaluation radiologique a été basée sur des clichés standards de l’épaule face. Ces mêmes clichés ont été utilisés pour évaluer le délai de consolidation. Le stade radiologique était évalué selon la classification de Neer [9] et l’évaluation fonctionnelle a été faite selon le score de Constant et Murley [10] absolu moyen qui a pu être calculé pour chaque patient.

### L'analyse statistique

D'abord une description globale des deux populations étudiées. Les comparaisons des moyennes ont été effectuées au moyen de test t de Student. Une analyse uni variée a été utilisée afin d'identifier les variables associées à l'indication de traitement chirurgical par plaque vissée comparée à l'indication de l'embrochage dans le traitement chirurgical des fractures du quart externe de la clavicule type II de Neer. Le seuil de 5% a été considéré comme statistiquement significatif. Les calculs ont été effectués en utilisant le logiciel SPSS dans sa version 15.0.

## Résultats

Vingt-six patients ont été étudiés, tous présentant une fracture distale de la clavicule type II de Neer, 12 patients ont été traités par plaque à crochet vissée et 14 patients par brochage transacromiale. Nous avons opéré 14 patients par plaque à crochet, 75% d'entre eux ont été victimes d'une chute sur l’épaule. Le groupe traité par brochage transacromiale comportait 12 patients dont cinq femmes et sept hommes, L’âge moyen global était de 31,6 (+/- 13,7) (20 à 45), le sex ratio (H/F) était de 1. L’étiologie du mécanisme lésionnel était une chute sur l’épaule le bras en adduction chez 13 patients soit 50% de notre population étudiée. Le délai moyen global de consolidation était de sept semaines (+/- 0,7) (6 à 9). Aucun patient des deux groupes n'a rapporté des douleurs au repos. En revanche, un patient du groupe brochage et trois patient du groupe plaque à crochet (16% des cas) ont présentés des douleurs à l’élévation de l’épaule. Le score douleur moyen sur l’échelle visuelle analogique est de 2 pour le groupe plaque vissée contre 4 pour le groupe brochage avec une différence significative entre les deux groupes (p= 0.033). Le score de Constant absolu moyen était de 86 (+/-10,4) pour le groupe brochage contre 90,92 (+/- 2,5) dans le groupe plaque vissée avec une différence significative entre les deux groupes (p= 0,04) (Tableau 1). Le délai moyen de consolidation était de 7,5 (+/- 0,7) semaines dans le groupe traité par plaque vissée, et de 7,1 (+/- 0,7) semaines dans le groupe traité par embrochage (p = 0,65). Un seul patient a nécessité une reprise chirurgicale du groupe brochage pour une pseudarthrose de la clavicule bénéficiant d'une greffe osseuse à partir de la crête iliaque et fixation par une plaque à crochet (Tableau 2). L'ablation de matériel est en moyenne de 5 + /- 2,3 mois pour le groupe brochage réalisé sous anesthésie locale avec une sédation contre 6 +/- 3.1 mois pour le groupe plaque à crochet réalisé sous anesthésie général de courte durée. Le temps opératoire moyen est de 35 min pour la plaque à crochet contre 45 minute pour le brochage avec une différence significative entre les deux techniques chirurgicales (p =0.038). Le saignement est estimé à 70cc pour le groupe brochage contre 100cc pour la plaque à crochet. Le nombre de complication est plus élevé dans le groupe brochage. Dans ce groupe nous avons 4 cas d'infection superficielle bien menée sous antibiothérapie par voie orale et 2 cas de recul de broche avec irritation de la peau. Dans le groupe plaque vissée nous avons deux complications; une ostéolyse de l'acromion et un 1 cas de conflit sous acromial disparu 2 mois après l'ablation de la plaque. Le coût du traitement dans notre centre peut être évalué à 150 euros pour un patient traité par embrochage et 280 euros pour un patient traité par plaque à crochet.

**Tableau 1:** Analyse démographique

	Plaque à crochet	Brochage transacromiale
Nombre de patients	14	12
Age moyen	34.2 (13,7)	29 (13,7)
***Sexe***		
Homme	8 (57.1%)	7 (58,3%)
Femme	6 (42.9%)	5 (41,7%)
***Mécanisme lésionnel***		
Chute sur l’épaule	8	5
Accident de circulation	6	7
Délai opératoire (jours)	1,5 + /-1,1	1,9 + /-1,7
Délai d'ablation de matériel (mois)	5,8 + /-0,7	4,4 + /-1,6
Score de constant moyen	91,92 (+/- 2,5)	86,4 (+/-10,4)

**Tableau 2:** Les complications post opératoire

	Plaque à crochet	Brochage transacromiale	P
Migration de matériels	---	02 cas	0.041
Infection	---	04 cas	0.035
Pseudarthrose	Aucun	01 cas	0.19
Conflits sous acromiale	01	---	0.03
Ostéolyse de l'acromion	01	---	0.255

## Discussion

Les fractures distales de la clavicule compte entre 10 et 26% et concerne essentiellement l'adulte jeune à la suite d'une chute sur l’épaule [9, 11]. [12] Dans les fractures Type II de Neer la séparation de la clavicule du complexe ligamentaire coracoclaviculaire ainsi que le jeu musculaire du membre supérieur sont d'autant des facteurs d'instabilité de la fracture ce qui impose la nécessité d'un traitement chirurgical, autrement le taux de pseudarthrose avoisine les 20% en cas de traitement orthopédique [1, 2]. La technique de choix dans le traitement de ce type de fracture reste controversée. Le moyen d'ostéosynthèse le plus couramment utilisé reste le brochage transacromiale avec des résultats radiologiques et fonctionnels satisfaisant avec un taux de consolidation qui atteint 95% avec peu de complications lié à la migration de broches [13]. Le brochage peut être réalisé en intra articulaire ou extra articulaire [14, 15]. Dans notre série l'utilisation du brochage est réalisée en intra articulaire chez 4 patients et extra articulaire chez 8 patients et nous a apporté une stabilisation satisfaisante dans les deux techniques. Cependant nous déplorons deux cas de recul de broches avec irritation cutanée et 4 cas d'infection superficielle et un cas de pseudarthrose qui a été repris par une plaque à crochet avec greffe cortico-spongieuse à partir de la crête iliaque.

Globalement les résultats fonctionnels restent satisfaisants. Nous recommandons le brochage bi cortical pour assurer une bonne tenue des broches afin de réduire le risque de migration [16]. Dans notre étude les résultats fonctionnels n'ont pas montré une différence significative entre les deux technique chirurgicale, de point de vue biomécanique [17] la plaque à crochet garde l'avantage d'une meilleur stabilisation par rapport au brochage avec une bonne fixation de la fracture tout en gardant l'intégrité de l'articulation acromio-claviculaire dans ces mouvement de rotation lors de l'abduction ou d'antépulsion de l’épaule [18, 19]. La stabilité assuré par la plaque a crochet permet une rééducation plus précoce par rapport au brochage sans restriction sur la mobilité de l'articulation avec un retour plus rapide à l'activité quotidienne.

**Figure 3:**
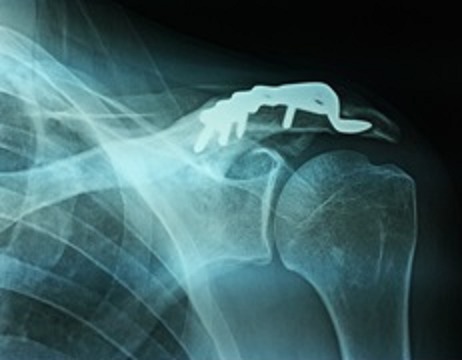
Femme de 26 ans opéré pour une fracture distale de la clavicule avec une image d'ostéolyse de l'acromion autour du crochet de la plaque vissée dernier contrôle radiologique à 9 mois

Un seul patient a rapporté un signe de conflit sous acromiale avec une abduction limité à 90°. Ceci peut être expliqué par l'emplacement du crochet de la plaque dans l'espace sous acromiale [20, 21]. Ce patient a noté une régression des signes huit semaines après l'ablation de la plaque avec une bonne mobilité articulaire. Nous rapportons le cas dune ostéolyse de l'acromion du au contact avec le crochet dont l’évolution s'est fait vers la recalcification de l'acromion 6 mois après l'ablation de la plaque vissée (Figure 3). Le meilleur moyen de prévenir ces deux complications est l'ablation de la plaque à crochet dès l'apparition de signes clinique et radiologique de consolidation [22].

## Conclusion

Le brochage transacromiale et la plaque à crochet reste deux moyens de fixation satisfaisant dans les fracture distales de la clavicule type II de Neer, ce pendant l'utilisation de la plaque à crochet garde l'avantage dans les fractures type II de Neer grâce à une bonne stabilité primaire avec une rééducation précoce, un taux de consolidation de 98% et un faible taux de complications à condition de réaliser l'ablation de matériel dès l'apparition de signes clinique et radiologique de consolidation.
